# ID 109 - Aplicação da Inteligência Artificial no Processo de Síntese de Evidências

**DOI:** 10.5327/2237-9622.2025.v34s1.109

**Published:** 2025-11-25

**Authors:** Graciela Paula do Nascimento Duque, Rosimary Terezinha de Almeida, Roberto Macoto Ichinose

**Keywords:** avaliação da tecnologia biomédica, inteligência artificial, revisão sistemática, processo de avaliação de tecnologias em saúde

## Abstract

**Introdução:**

No processo de Avaliação de Tecnologias em Saúde (ATS), a síntese de evidências é uma etapa laboriosa e que requer equipe qualificada. Na realidade dos Núcleos de Avaliação de Tecnologias em Saúde Hospitalares (NATS-H), os maiores desafios são a disponibilidade de pessoal e sua qualificação. Nesse cenário, a sustentabilidade da ATS nesses núcleos requer novas abordagens que otimizem o trabalho da equipe. As técnicas de Inteligência Artificial (IA) têm sido aplicadas para propiciar a automatização ou semiautomatização de etapas do processo de ATS, buscando reduzir o esforço humano na elaboração de síntese de evidências, conferindo melhor gerenciamento do tempo gasto no processo. Nesta perspectiva, o trabalho buscou identificar e organizar ferramentas de IA que podem contribuir nas diferentes etapas de elaboração de síntese de evidências.

**Método:**

A identificação das ferramentas de IA foi realizada mediante levantamento semiautomatizado no site Google Academics, por meio da criação do alerta para o termo:, no período de novembro de 2023 a agosto de 2024. Adotou-se o Google Academics como fonte de informação por englobar diferentes áreas do saber e pela agilidade na compilação de material bibliográfico em relação às bases convencionais. O processo de identificação ocorreu, inicialmente, pela triagem do título das referências recebidas diariamente por meio do alerta. Em uma segunda etapa, foram selecionadas as publicações relevantes após a obtenção do texto completo. Dada a especificidade e a dinâmica do desenvolvimento acelerado do uso de IA no processo de automatização, optou-se por priorizar os artigos de revisão.

A organização das ferramentas de IA identificadas nas revisões ocorreu a partir de uma classificação apresentada em um trabalho seminal sobre automatização do processo de revisão, que agrupou as diversas tarefas de elaboração de síntese de evidências em cinco grupos: preparação, recuperação, avaliação, síntese e relatoria.

**Resultados:**

Na etapa de pré-seleção desta pesquisa, foram obtidos 91 artigos publicados entre os anos 2019 e 2024, entre os quais 29 citavam ferramentas de IA na elaboração de síntese de evidências, sendo que seis destes foram priorizados por se tratar de artigos de revisão. Ao todo foram identificadas 101 ferramentas de IA com uso nos cinco grupos de tarefas, algumas de uso concomitante, assim distribuídas: preparação (51 ferramentas); recuperação (11 ferramentas); avaliação (39 ferramentas); síntese (46 ferramentas); e relatoria (16 ferramentas).

**Conclusão:**

O levantamento realizado neste estudo aponta para a existência de uma diversidade de ferramentas de IA para uso nas diferentes tarefas de síntese de evidências possibilitando a elaboração de relatórios de ATS de forma a mantê-los atualizados com maior agilidade, redução do volume de trabalho e economia de tempo.

Contudo, a viabilidade de uso dessas ferramentas em Nats-H deve ser investigada levando-se em conta os custos, os recursos físicos necessários, a necessidade de capacitação da equipe de trabalho, além da usabilidade dessas ferramentas para que as demandas de ATS locais possam ser atendidas de forma concisa e em tempo favorável.

